# Impact of Gut Microbiota and Microbiota-Related Metabolites on Hyperlipidemia

**DOI:** 10.3389/fcimb.2021.634780

**Published:** 2021-08-19

**Authors:** Xiaokang Jia, Wen Xu, Lei Zhang, Xiaoyan Li, Ruirui Wang, Shuisheng Wu

**Affiliations:** ^1^College of Pharmacy, Fujian University of Traditional Chinese Medicine, Fuzhou, China; ^2^Centre of Biomedical Research & Development, Fujian University of Traditional Chinese Medicine, Fuzhou, China; ^3^The Eighth Affiliated Hospital of Sun Yat-sen University, Shenzhen, China; ^4^Shanghai Innovation Center of TCM Health Service, Shanghai University of Traditional Chinese Medicine, Shanghai, China

**Keywords:** gut microbiota, hyperlipidemia, BAs, LPS, SCFAs

## Abstract

Hyperlipidemia, defined as the presence of excess fat or lipids in the blood, has been considered as a high-risk factor and key indicator of many metabolic diseases. The gut microbiota has been reported playing a vital role in regulating host lipid metabolism. The pathogenic role of gut microbiota in the development of hyperlipidemia has been revealed through fecal microbiota transplantation experiment to germ-free mice. The effector mechanism of microbiota-related metabolites such as bile acids, lipopolysaccharide, and short-chain fatty acids in the regulation of hyperlipidemia has been partially unveiled. Moreover, studies on gut-microbiota-targeted hyperlipidemia interventions, including the use of prebiotics, probiotics, fecal microbiota transplantation, and natural herbal medicines, also have shown their efficacy in the treatment of hyperlipidemia. In this review, we summarize the relationship between gut microbiota and hyperlipidemia, the impact of gut microbiota and microbiota-related metabolites on the development and progression of hyperlipidemia, and the potential therapeutic management of hyperlipidemia targeted at gut microbiota.

## Introduction

With the improvement of living standards and the aging of the global population, the incidence of metabolic diseases such as obesity, diabetes, arteriosclerosis, stroke, coronary heart disease, and myocardial infarction is increasing rapidly (Mangiafico et al., 2013; Ezzati et al., 2015; Townsend et al., 2015; Benjamin et al., 2018). Notably, dyslipidemia or hyperlipidemia is a common feature among these chronic diseases. Hyperlipidemia is characterized by high levels of total cholesterol (TC), low-density lipoprotein cholesterol (LDL-C), and triglycerides (TG) and a low level of high-density lipoprotein cholesterol (HDL-C) (Mitchell et al., 2016; Deng et al., 2019). The etiology of hyperlipidemia is determined by a combination of genetic and environmental factors, such as diet, lifestyle, and even geographic location (Aguilar-Salinas et al., 2002), but the underlying mechanism remains unclear. The incidence and mortality rates of hyperlipidemia and its complications are also increasing rapidly, accounting for nearly half of the deaths worldwide (GBD 2017 Disease and Injury Incidence and Prevalence Collaborators, 2018). Early treatment of hyperlipidemia can effectively prevent the occurrence of related diseases (Gordon et al., 1989; Baigent et al., 2005; Triglyceride Coronary Disease Genetics Consortium and Emerging Risk Factors Collaboration, 2010).

Recent studies have shown that gut microbiota is a critical environmental factor in the regulation of body metabolism, contributing to the occurrence and development of chronic diseases, such as obesity, diabetes, and atherosclerosis (Janssen and Kersten, 2015; Brahe et al., 2016; Brial et al., 2018). Gut microbiota represents the huge community of bacteria residing in the gastrointestinal tract. The total amount of gut microbiota genes is 100 times that of human genes and is known as the “second genome” of humans (Moeller et al., 2012; Komaroff, 2018). Gut microbiota serves several functions in host metabolism, such as supplementing body nutrition (Du Toit, 2016), promoting immunity (Bender, 2020), and protecting the intestinal barrier (Shin and Kim, 2018). Given its regulatory role in host lipid metabolism, gut microbiota has been highly associated with hyperlipidemia and related diseases (He et al., 2016). Attribute to the germ-free animal model, the casual role of gut microbiota it the development has been established. Moreover, gut-microbiota metabolites such as short-chain fatty acids (SCFAs), lipopolysaccharide (LPS), and bile acids (BAs) have been discovered in the occurrence and development of hyperlipidemia.

In the present paper, we propose that gut microbiota is the key to the development of hyperlipidemia and related chronic diseases. Understanding the interaction between gut microbiota and hyperlipidemia is important to prevent and improve hyperlipidemia-related diseases.

## Relationship Between Gut Microbiota and Hyperlipidemia

### Dysbiosis of Gut Microbiota in Individuals With Hyperlipidemia

The disturbance of lipid metabolism could induce changes the intestinal environment, which could induce the dysbiosis of internal microflora (Fukuda and Ohno, 2014). The development of high-throughput technology enables us to deepen the understanding of the composition of different ecosystems in the human gut and the role of metabolites.

It has been reported that individuals with hyperlipidemia exhibit disturbed structure and function of gut microbiota. Children and adolescents with hyperlipidemia show low levels of fecal acetate, butyrate, and propionate, which are associated with SCFA-producing bacteria, such as those from families Lachnospiraceae and Ruminococcaceae and genera *Akkermansia*, *Bacteroides*, *Roseburia*, and *Faecalibacterium* (Gargari et al., 2018). A randomized controlled trial has shown that, compared with the feces of healthy individuals, the feces of patients with metabolic syndrome(charaterized by hyperlipidemia) contain lower abundance of potential probiotics such as *Bifidobacterium*, *Lactobacillus*, *Faecalibacterium prausnitzii* and *Roseburia* but higher abundance of LPS-producing bacteria (*Escherichia coli* and *Enterobacter cloacae*) (Moreno-Indias et al., 2016). Studies based on the conventionalization of germ-free (GF) and conventionally raised (CV) animals recognize the special role of the intestinal microbiota in host metabolism. Compared with healthy mice, diabetic db/db mice with lipid-metabolism disorder have lower levels of butyrate-producing bacteria (e.g., *Fecalibacterium prausnitzii*, *Eubacterium rectale*, and *Roseburia intestinalis*) and higher levels of opportunistic pathogens (e.g., *Clostridium hathewayi*, *Clostridium ramosum*, and *Eggerthella lenta*) (Qin et al., 2012; Wang et al., 2020). The feces of rats with hyperlipidemia contains significantly more LPS-producing bacteria (e.g., *Bilophila* and *Sutterella*) and mucosa-damaging bacteria (*Bilophila* and *Akkermansia muciniphila*) (Song et al., 2017). Hyperlipidemia is also associated with microbial structure and function in the ileum, which can transform glycine or taurine-conjugated BAs into unconjugated BAs (Huang et al., 2019). Other studies have shown that the structure of gut microbial in hyperlipidemia rats is changed, which reduces the excretion of BAS in the cecum (Ma et al., 2018; Pan et al., 2018; Han et al., 2019). The gut microbial trimethylamine N-oxide (TMAO) pathway is also closely linked to host cholesterol and BA metabolism, TMAO can reduce the expression of the key enzyme CYP7A1 in bile acid synthesis, reduce the bile acid pool, inhibit cholesterol transport, and cause the accumulation of cholesterol in the cell (Pathak et al., 2020).

At present, a clear consensus about the imbalances in gut microbiota composition under hyperlipidemia remains to be achieved, which may attributed to the fact that gut microbiota shares similar functions across species (Koh and B?ckhed, 2020). At the metabolite level, individuals with hyperlipidemia show low levels of SCFAs, high levels of LPS and TMAO, and changed BA pool.

### Gut Microbiota Regulates Lipid-Metabolism Homeostasis and Contributes to the Development of Hyperlipidemia

Normal gut microbiota is essential in maintaining lipid-metabolism homeostasis (Fu et al., 2015; Chen et al., 2019). Compared with CV mice, GF mice show a lower level of TG in the adipose tissue and the liver but a higher level of TG in the circulatory system (Velagapudi et al., 2010). When fed with high-fat, high-carbohydrate Western diet, GF mice have less hypercholesterolemia and more cholesterol excretion in the liver and feces (Rabot et al., 2010). After a single gavage of olive oil in GF and conventional mice, GF mice show higher serum TG and longer metabolism time of TG (Bäckhed et al., 2007). The exposure of mice to a low-dose endotoxin (LPS) significantly increases the level of serum TG (Hardardóttir et al., 1995). Bacteria with LPS-producing ability could increase serum endotoxin load and aggravate inflammatory response, leading to fat accumulation (Na and Liping, 2013). These findings revealed that gut microbiota regulates lipid-metabolism homeostasis and contributes to the development of hyperlipidemia.

### The Underlying Mechanism Between Gut Microbiota and Lipid Metabolism

**Figure 1:**
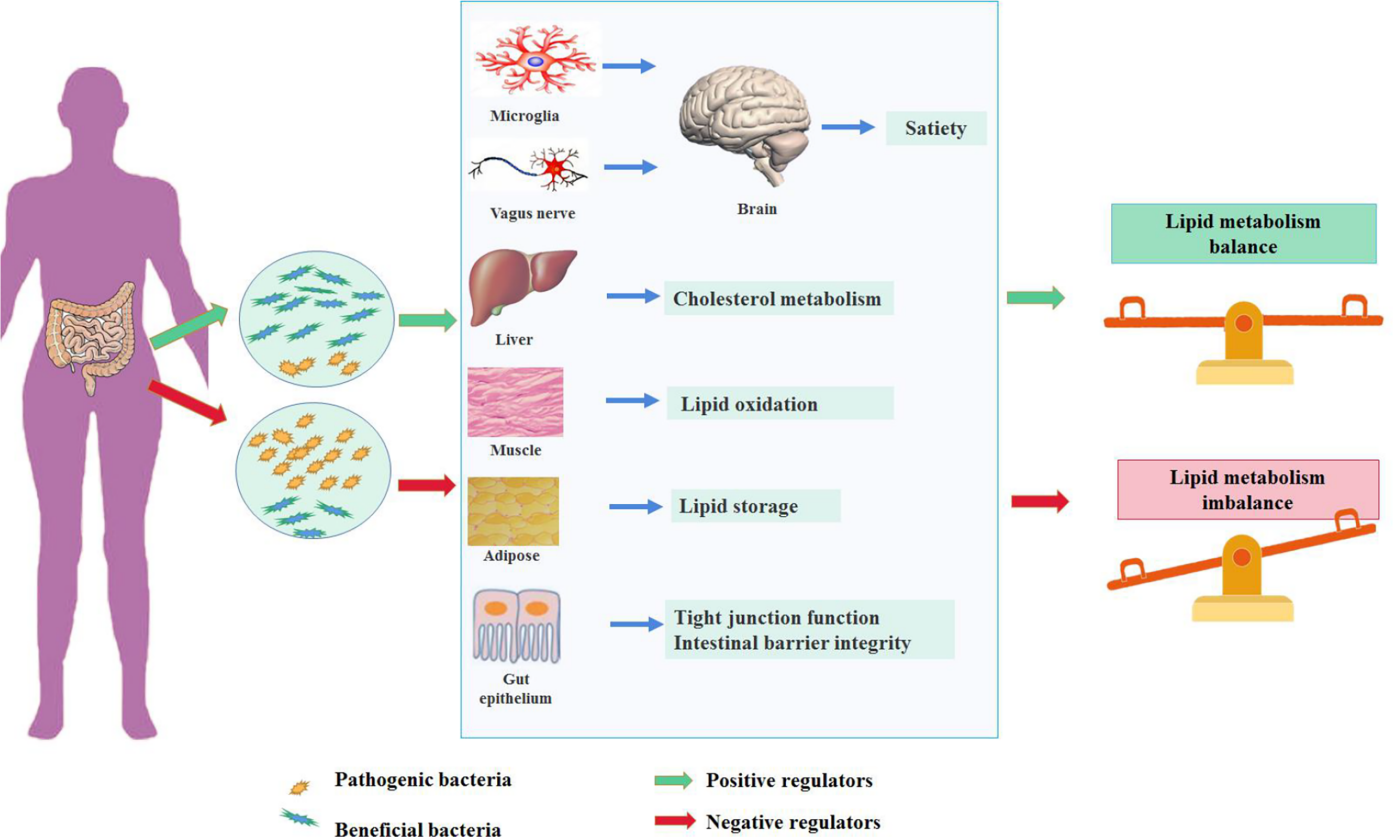
The impact of gut microbiota in the development of hyperlipidemia.

Diet is the main factor affecting the diversity of intestinal bacteria. The Western diet represented by high fat and high sugar can change the composition of intestinal flora and directly promote the occurrence of lipid metabolism disorder. Several pathways have been proposed in the regulation of lipid metabolism mediated by gut microbiota (**Figure 1**). Gut microbiota and the related metabolites could protect or disrupt the integrity of the intestine, thereby affecting the function of peripheral tissues (Qi et al., 2021). Gut microbiota can regulate the brain–gut axis through immune response and the vagus nerve. Imbalances in gut microbiota can lead to the proliferation of potential pathogenic bacteria, affect immune homeostasis, and induce a pro-inflammatory local immune response (Sanz and Moya-Pérez, 2014). This phenomenon is accompanied by inflammatory cytokine and adipokine production [e.g., tumor necrosis factor-α, interleukin (IL)-6 and IL-1β, and leptin] (Zeyda and Stulnig, 2007; Kanneganti and Dixit, 2012). At the same time, the increase in intestinal permeability caused by a high-fat diet (HFD) can aggravate this situation (de La Serre et al., 2010; Muccioli et al., 2010; Lim et al., 2015). In addition to its effects on peripheral immune cells, gut microbiota can also reportedly regulate immune cells (microglia) located in the brain, causing microglia activation and neuroinflammation and affecting the hypothalamic neurons that regulate appetite. Consequently, food intake is reduced and energy consumption is increased (D’Mello et al., 2015; Kim et al., 2019). Another study has shown that vagotomy in animal models results in decreased anorexia hormone signaling and subsequently increased food intake and body-weight gain (Perry et al., 2016a). The metabolites produced by gut microbiota can activate enteroendocrine cells to release intestinal hormones and can directly interact with the enteric nervous system and its innervating vagus nerve. The generated local signals can then be transmitted through sensory neural circuits to the brain regions involved in eating behavior, affecting appetite and satiety (Mulders et al., 2018). Gut microbiota can also change the integrity of intestinal epithelial cells and the intestine, regulate cholesterol metabolism in the liver, promote lipid oxidation in muscles, and regulate lipid storage in adipose tissue, thereby regulating lipids’ metabolic balance (Bäckhed et al., 2004; Rohr et al., 2020).

A balanced gut microbiota (the microorganisms of the gut microbiota stay in the mutualistic symbioses and create a stable intestinal microsystem) contributes to the maintenance of lipid homeostasis, referred to various tissues and organs, including mediating satiety in the brain through microglia and vagus nerves, regulating cholesterol metabolism in the liver, promoting lipid oxidation in the muscle and energy storage in the adipose tissue, and maintaining the integrity of the intestinal barrier. In contrast, the dysbiosis of gut microbiota could disturb the lipid metabolism through mechanisms mentioned above.

## Metabolites of Gut Microbiota and Hyperlipidemia

Gut microbiota can ferment food residues, produce a large number of metabolites, and regulate biological functions in the human body (Flint et al., 2012; Hood, 2012). Host health is closely related to microbial metabolites, including SCFAs (Bartolomaeus et al., 2019), BAs (Wahlström et al., 2016), LPS, TMAO (Wang et al., 2011; Wu et al., 2019), polyphenols, polyamines, indoles, vitamins, and amino acids (Xu et al., 2016; Yan et al., 2016; Chittim et al., 2019). The present study emphasizes the relationship of hyperlipidemia to BAs, LPS, and SCFA.

### BAs and Hyperlipidemia

**Figure 2:**
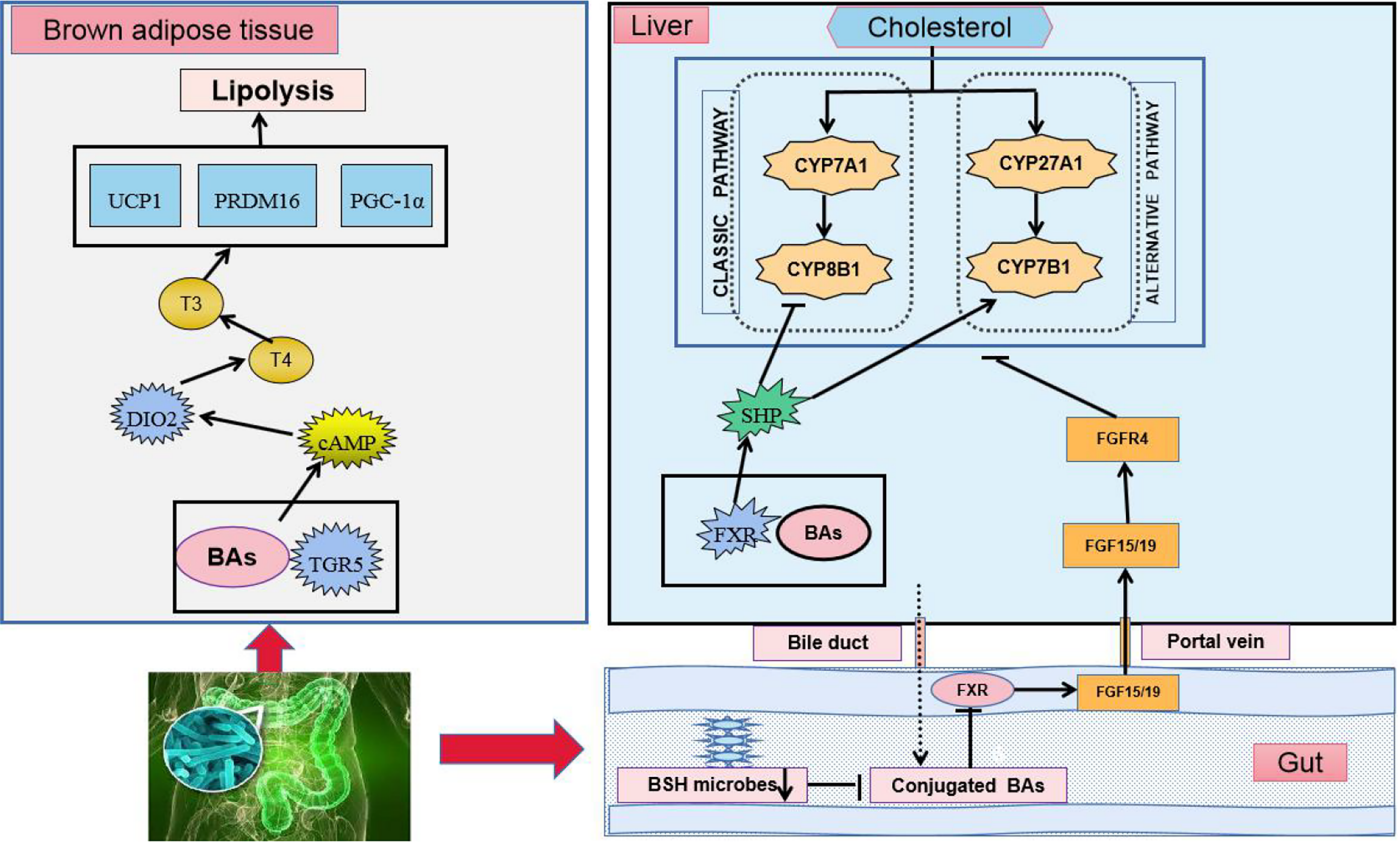
BAs regulate TGR5 and FXR in lipid-metabolism disorder. Cholesterol regulates the synthesis of BAs and completes the circulation of the liver and intestine through two ways.

Primary BAs are synthesized by cholesterol in the liver and enter the intestinal tract to participate in lipid digestion (LaRusso et al., 1974; Swann et al., 2011). The majority of BAs are reabsorbed in the ileum and transported to the liver through the portal vein, whereas the minority enters the large intestine (Russell, 2003). Different types of BAs exert different regulatory effects on lipid metabolism. Gut microbiota can regulate lipid metabolism by balancing the BA pool and composition. Under the action of gut microbiota in the terminal ileum and colon, primary BAs are converted into secondary BAs by microbial-related bile-salt hydrolase (BSH) and 7-dehydroxylase (Russell and Setchell, 1992; Ridlon et al., 2006). In patients with hyperlipidemia, the synthesis of total BAs is increased, the BA pool is enlarged, and the ratio of primary to secondary BAs in the intestine is significantly changed (Einarsson et al., 1974; Podda and Zuin, 1985). In mice treated with antibiotics, BA secretion in the liver is increased three times, whereas that in excreta is significantly decreased (Miyata et al., 2009). Antibiotics also significantly increase hepatic BA secretion while reduce BA excretion (Thomas et al., 2010). BAs regulate lipid metabolism and energy homeostasis by activating G-protein-coupled receptor-5 (TGR5) and farnesoid X receptor (FXR) (**Figure 2**) (Watanabe et al., 2006). As an agonist of TGR5, BAs can directly activate TGR5 and its downstream energy-consumption-related signal pathways in brown adipocytes, thereby promoting the energy consumption of adipose tissue and muscle in mice (Broeders et al., 2015). FXR also participates in the regulation of BA enterohepatic circulation and lipid metabolism, which exerts a tissue-specific effect. BAs can inhibit intestinal FXR signaling, increase fibroblast growth factor 15/19 (FGF15/19) production, and affect the secretion of cell-surface fibroblast growth factor receptor 4, ultimately promoting BA synthesis and decrease cholesterol levels (Vergnes et al., 2013). BAs can also inhibit adipogenesis by activating FXR in the liver (Cariou et al., 2011). Compared with intestinal-specific FXR knockout mice, liver-specific FXR gene knockout mice show poor ability to maintain blood lipid. Intestine-specific FXR gene knockout can reduce liver fat accumulation, inhibit intestinal FXR expression, reduce hepatic gluconeogenesis, and improve glucose and lipid-metabolism disorder in mice fed with HFD (Jiang et al., 2015; Schmitt et al., 2015).

BAs also can shape the structure of gut microbiota. The biotransformation of BAs plays a certain role in the detoxification of gut microbiota so that bacteria can avoid being killed by BAs (Ridlon et al., 2014). When combined with phospholipids on the bacterial cell membrane, BAs can exert a destructive role in bacterial cells, resist bacterial adhesion, and neutralize endotoxins; high concentrations of conjugated BAs exert a direct antibacterial effect (Fouts et al., 2012). Meanwhile, different bacteria have different degrees of sensitivity to BAs. HFD rich in saturated fatty acids can promote BA secretion, thereby changing the living environment of bacteria. Consequently, the gut-microbiota structure becomes changed, the growth of some potential pathogens in the intestine is promoted, and the lipid metabolism of the host is adversely affected (Devkota et al., 2012).

In brown adipose tissue, the activation of TGR5 induces the intracellular accumulation of cAMP, followed by the downstream activation of its signaling pathways, such as the activation of DIO2. DIO2 converts the prohormone T4 into the active hormone T3 and increases metabolic rates. Consistent with this feature, the activation of TGR5 enhances the expression of a series of downstream genes related to the energy expenditure of TGR5 including D2, UCP1, PRDM16, and PGC-1α *in vivo* and *in vitro*. In the liver, BAs combine with FXR to activate the FXR-SHP signaling pathway. In the intestine, BSH enzymes are produced in intestinal microbes and function to hydrolyze conjugated BAs into unconjugated ones. The suppression of BSH microbes results in the accumulation of conjugated BAs, the downregulation of FXR, the inhibition of the FXR-FGF15/19 signaling pathway, and the regulation of cholesterol synthesis.

cAMP: cyclic adenosine monophosphate, DIO2: deiodinase, iodothyronine, Type II, T3: tri-iodothyronine, T4: thyroxine, UCP: uncoupling protein, PRDM16: PR domain-containing 16, PGC1α: peroxisome proliferator-activated receptor gamma coactivator 1 alpha, CYP7A1: cholesterol 7α-hydroxylase, CYP8B1: sterol 12α-hydroxylase. CYP27A1: steroid 27-hydroxylase, CYP7B1: oxysterol 7α-hydroxylase, SHP: small heterodimer partner.

### LPS and Hyperlipidemia

**Figure 3:**
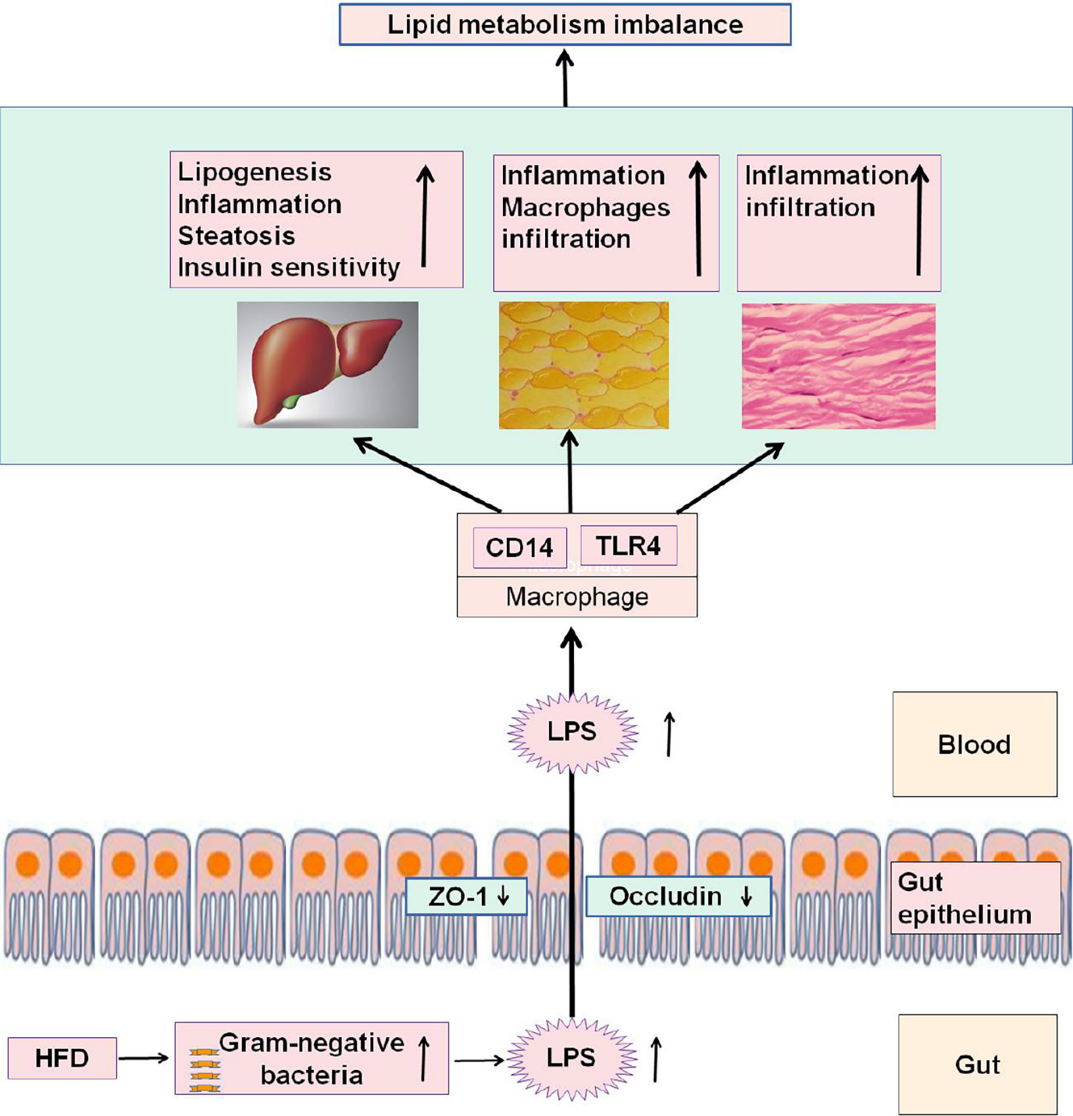
HFD feeding changes gut microbiota, promotes metabolic endotoxemia, and triggers the development of metabolic disorders.

LPS, also known as endotoxin, is an essential structural compound of the outer membrane of Gram-negative bacteria (Raetz and Whitfield, 2002). After the death and lysis of bacteria, LPS is released into the intestinal environment and enter the circulatory system through the “leaky gut” (an increased intestinal permeability) (Rietschel et al., 1994). High concentrations of LPS could induce endoxemia characterized by serious nonspecific immune response. LPS also promotes the redistribution of nutrients in the host defense during the acute phase, and blood lipids play a protective role by neutralizing the level of inflammatory cytokines (Beigneux et al., 2000). HFD, fatty acid exposure, and low-dose LPS could induce metabolic endotoxemia, which features chronic inflammation, insulin resistance, and lipid-metabolism disorder (Harris et al., 1990; Feingold et al., 1995; Erridge et al., 2007). However, supplementing HFD with antibiotics to reduce the production and blood inflow of intestinal LPS could improve the above symptoms of metabolic disorders (Cani et al., 2008). The systemic inflammatory responses induced by HFD are primarily attributed to the increase in the blood level of LPS (Kim et al., 2014). Serum LPS levels are higher in patients with obesity and mice with HFD-induced obesity than in normal controls (Ghanim et al., 2009). The HFD-induced dysbiosis of gut microbiome can damage the intestinal mucosal barrier and trigger the release of LPS into the blood (Hersoug et al., 2016). LPS can promote the infiltration of inflammatory cells and the expression of inflammatory factors in adipose tissue (Caesar et al., 2012). Furthermore, mice lacking toll-like receptors 4 receptor and cluster-of-differentiation 14 (the receptor of LPS) are resistant to HFD- or LPS-induced hyperinsulinemia, insulin resistance, and steatosis (Cani et al., 2007). These results confirm that HFD feeding changes gut microbiota, promotes metabolic endotoxemia, and triggers the development of metabolic disorders (**Figure 3**).

In the intestine, HFD feeding can increase Gram-negative bacteria, increase the LPS content, and decrease the tight-junction proteins ZO-1 and occludin, thereby affecting the permeability and integrity of intestinal epithelial cells. LPS entering the blood can be recognized and bound by LPS-binding protein and then form an LPS/CD14 complex with the CD14 of mononuclear macrophages, leading to a series of intracellular reactions through TLR-4. Consequently, inflammatory factors are expressed and released, thereby reducing the ability of the liver, adipose, and muscle to regulate lipid metabolism, causing the body's lipid-metabolism disorder.

TLR4: toll-like receptors 4 receptor, CD14: cluster-of-differentiation 14, ZO-1:zonula occluden 1

### SCFAs and Hyperlipidemia

**Figure 4:**
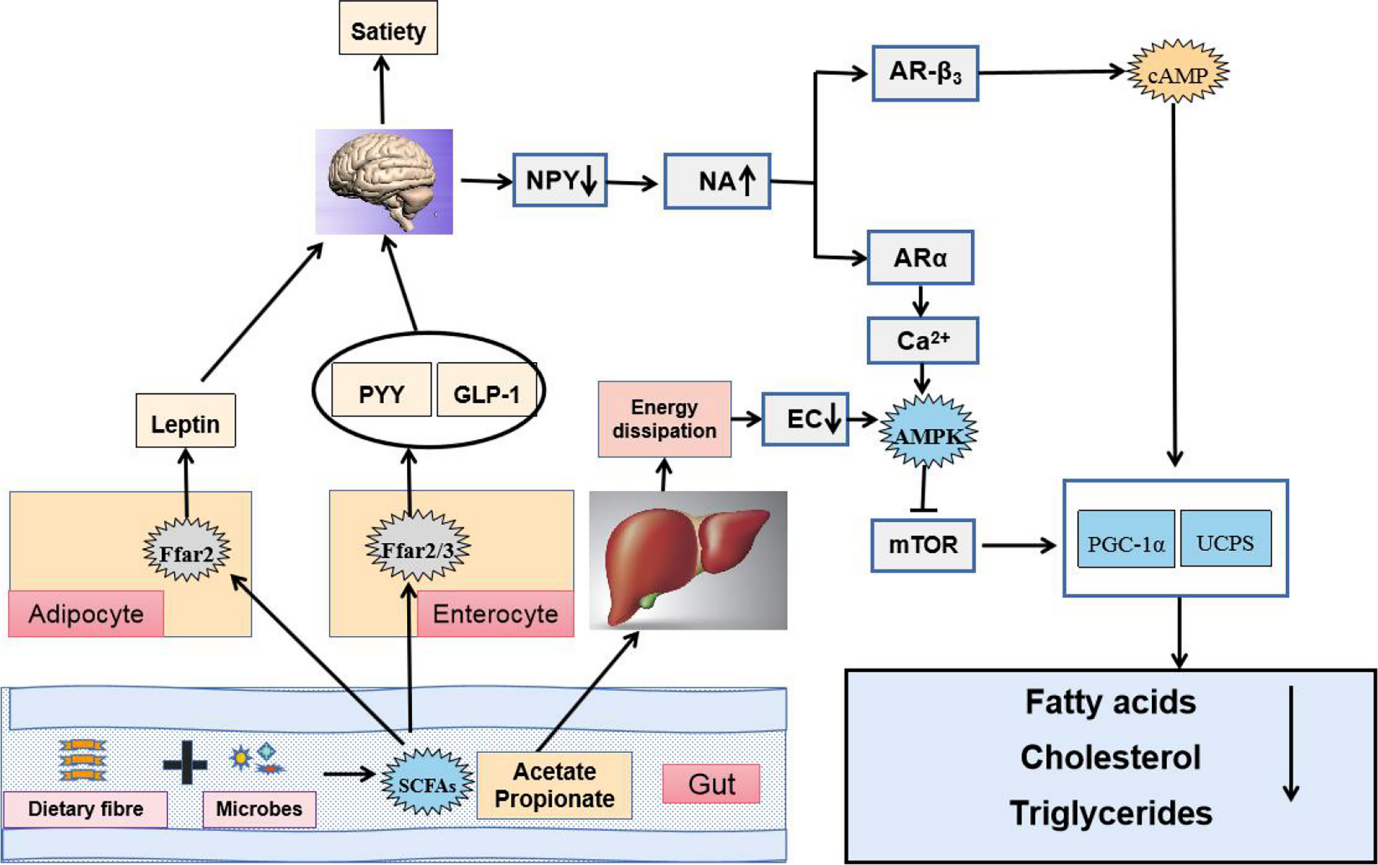
SCFAs regulate energy metabolism through the gut–brain axis and the AMPK pathway to improve dyslipidemia.

SCFAs, fermented by gut microbes from dietary fiber, are primarily composed of acetic, propionic, butyric, isobutyric, valeric, and isovaleric (Bergman, 1990; Miller and Wolin, 1996). Diverse genus such as *Bifidobacterium*, *Lactobacillus*, *Bacteroides*, and *Faecalibacterium* can produce SCFAs (Hold et al., 2003; Chambers et al., 2015; Koh et al., 2016). SCFAs are quickly and efficiently absorbed in the colon and cecum, whereas only 5%–10% of SCFAs are excreted with feces (Canfora et al., 2015). SCFAs can be utilized as direct energy source by intestinal epithelial cells and liver (McNeil, 1984). The liver can absorb about 30% of SCFAs from the portal vein, which can promote lipid production and cholesterol synthesis (Royall et al., 1990; Bloemen et al., 2009; den Besten et al., 2013). Notably, SCFAs (acetate, butyrate, and propionate) can regulate lipid metabolism in the body (Roediger, 1980; Ardawi and Newsholme, 1985; Cummings et al., 1987). Once produced, SCFAs are readily absorbed and utilized in the body. Butyrate is largely utilized by the colonic epithelium as an energy source, and propionate is primarily utilized by the liver, whereas a significant amount of acetate enters systemic circulation and reaches peripheral tissues (Marques et al., 2018). SCFAs could act as a regulating signal of energy metabolism through the gut–brain axis and the adenosine monophosphate-activated protein kinase (AMPK) pathway to improve dyslipidemia ( Dalile et al., 2019; Xiao et al., 2019). By binding to G-protein-coupled receptor, SCFAs could induce the release of hormones, which could increase satiety and reduce food intake. Moreover, elevated leptin level could regulate lipid metabolism through signal regulation. The absorption of acetate and propionate by the liver also activates the AMPK pathway and inhibits fatty acid, cholesterol, and TG synthesis (Samuel et al., 2008; Tazoe et al., 2008; Lin et al., 2012). An animal study has shown that mice with insulin resistance induced by an HFD results in significantly increased amount of acetate in the body, thereby activating the parasympathetic nervous system, promoting glucose-stimulated insulin secretion, and increasing the appetite, and eventually leading to imbalanced lipid metabolism (Perry et al., 2016b). Meanwhile, the combination of butyrate and propionate with GPR43, a G-protein-coupled receptor (GPCR), can inhibit the decomposition of fatty acids, promote the secretion of the best known fat hormone leptin, increase energy release, and inhibit the synthesis of fat cells, which reduce lipid metabolism in mice; these effects disappear in GPR43-knockout mice (Ge et al., 2008). The addition of exogenous sodium butyrate could improve insulin sensitivity in HFD-induced insulin resistance mice by promoting mitochondrial function, peroxisome proliferator-activated receptorγcoactivator-1 (PGC-1) expression, and fatty acid oxidation (Gao et al., 2009). However, some studies have reported that butyrate plays an opposite role in lipid metabolism, which requires further discussion (Huang et al., 2017). Thus, SCFAs can regulate energy metabolism to improve dyslipidemia (**Figure 4**).

The brain could sense the microbial metabolites not only via release of hormones such as PYY, GLP-1 and LEP after SCFAs binding to G-protein-coupled receptors (GPCRs) GPR41 (also known as Ffar3) and GPR43 (also known as Ffar2) in fat cells and enteroendocrine L-cells but also through faster neuronal signaling, which decreased food intake, suppressed the activity of orexigenic neurons that expressed neuropeptide Y in the hypothalamus and raised the sympathetic activity. Elevated plasma contents of NA induced gene expression of uncoupling proteins (UCPs) capable of dissipating the proton gradient to generate heat instead of ATP synthase. Additionally, acetate and propionate taken up by the liver were used as substrates for lipogenesis and gluconeogenesis, which increased energy dissipation. As a result, energy charge (EC) lowered in the liver and 5′AMP-activated protein kinase (AMPK) became activated because AMP binding to γ subunit of AMPK promoted a conformational change and exposed the catalytic domain on the α subunit. Phosphorylated AMPK acted on downstream target proteins and showed functions of inhibiting synthesis of fatty acids, cholesterol and triglycerides, and activating fatty acid uptake.

## Treatment of Hyperlipidemia Targeted at Gut Microbiota

In the clinical setting, the medication treatment for hyperlipidemia is delivered largely through four classes of drugs: cholesterol-lowering agent (statins, cholesterol absorption inhibitors, probucol, and bile acid sequestrants) (Insull, 2006), TG-lowering agent (fibrates, niacin, and omega-3 fatty acid) (Chapman et al., 2011), some emerging therapeutic agents [lomitapide (Cuchel et al., 2013), mipomersen (Raal et al., 2010), alirocumab, and evolocumab (Stein et al., 2012)], and natural herbal medicines. Clinical studies have found that statins can affect gut microbiome during treatment, but few studies have focused on the effect of other lipid-lowering drugs on the gut microbiome of the intestine, which is also a limitation in this field. Given the evidence supporting the role of intestinal microbiota in hyperlipidemia, understanding how we can beneficially modify microbiota has become increasingly important. This section summarizes the main preventive and therapeutic interventions modulating gut microbiome (**Table 1**).

**Table 1:** Treatment of hyperlipidemia targeted at gut microbiota.

Therapeutic method	Names	Research subjects	Changes in intestinal microflora, metabolites, or related factors		Principal results
			Increased abundance	Decreased abundance	
**Prebiotics**	Inulin (Li et al., 2019)	Mice with type-2 diabetes (T2DM)	Cyanobacteria and *Bacteroides*	Ruminiclostridium-6, Deferribacteres, and Tenericutes	Reduced FBG, glycated hemoglobin, blood lipid, LPS, and IL-6
	Chicorium intybus (Liu et al., 2017)	Healthy adults	Breath hydrogen and SCFAs	Not listed	Increased plasma GLP-1 and PYY concentrations and lowered hunger rates
	Orange juice (Fidélix et al., 2020)	Ten women	*Lactobacillus* spp., *Akkermansia* spp., and *Ruminococcus* spp.	Not listed	Modulated intestinal microbiota and improved glycemia and lipid profiles
	EGCG3″Me (Zhang et al., 2018)	High-fat-diet (HFD) mice	Bacteroidetes	Firmicutes/Bacteroidetes	EGCG3″Me shows a weight-reducing effect and ameliorates the HFD-induced gut dysbiosis
	*Green tea extract combined with isomalto-oligosaccharides* (Singh et al., 2017)	HFD mice	Prevotella/Bacteroides	Firmicutes/Bacteriodetes	Prevented HFD-induced adiposity and lipid accumulation in liver and muscle, as well as normalized fasting blood glucose, insulin, glucagon, and leptin levels
**Probiotics**	Fermented soy milk (Kim et al., 2014)	HFD Rat	Reduced SREBP-dependent cholesterol and TG synthesis in the liver and enhanced adiponectin signaling and PPARα-induced expression of genes involved in TG-rich lipoprotein clearance		Lowered hepatic lipids and serum TG and FFAs and elevated HDL-C
	*Lactobacillus reuteri* 263 (Huang et al., 2015)	Hamster model of hyperlipidemia	Not listed	Not listed	Increased HDL-C and decreased TC, TG, LDL-C, and LDL-C/HDL-C ratio
	Probiotic mix (Sharma et al., 2016)	Hamster model of hyperlipidemia	Reduced expression of intestinal NPC1L1 and MTTP		Reduced TC, TG, and fatty acids
	*Lactobacillus fermentum* 5898 (Yadav et al., 2019)	Fed cholesterol-enriched diet rat	Repressed oxidative stress created by excess cholesterol by increasing the antioxidative enzyme activities and by decreasing lipid peroxidation		Reduced TG, TC, and LDL-C, and raised HDL-C
	*Lactobacillus casei* YBJ02 (Qian et al., 2019)	HFD mice	*Bacteroides* and *Akkermansia*	Firmicutes	Reduced TG, TC, and LDL-C, and raised HDL-C
	Three *Lactoplantibacillus plantarum* strains (Guerrero-Bonmatty et al., 2021)	LDL-C and other blood lipid parameters of hypercholesterolemic subjects	Not listed	Not listed	Reduced TC and LDL-C
	*Lactobacillus plantarum Q180* (Park et al., 2020)	Healthy adults	*R. Bromii*, *K. alysoides*, *B. intestinihominis*, and *F. plautii*	*Escherichia coli*, *Clostridium* sp., *Bacteroides fragilis*, *Enterococcus faecalis*, and *Proteus* sp.	Decreased LDL-C, TG, and apolipoprotein (Apo) B-100 levels and decreased areas under the curve of TG, ApoB-48, ApoB-100, total indole, and phenol levels
FMT	Fecal bacteria from healthy mice (Sun et al., 2019)	ob/ob mice	Lachnospiraceae, *Clostridium*, and Butyrate	Not listed	Improved glucose and lipid metabolism and alleviated hepatic steatosis
	Theabrownin FMT (Huang et al., 2019)	HFD mice	Bacteroidia	*Lactobacillus*, *Bacillus*, *Streptococcus*, and *Lactococcus*	Reduced weight gain and serum TC and TC
	Resveratrol FMT (Lai et al., 2018)	HFD mice	*Bacteroides*, Lachnospiraceae_NK4A136_group, *Blautia*, *Lachnoclostridium*, *Parabacteroides*, and *Ruminiclostridium*_9	Not listed	Modulated lipid metabolism, stimulated development of beige adipocytes in WAT, reduced inflammation, and improved intestinal-barrier function
	Autologous FMT (Rinott et al., 2021)	Abdominally obese or dyslipidemic participants	*Akkermansia muciniphila*	*Lactobacillus ruminis*	Compared with control-diet aFMT, significantly prevented weight regain and resulted in better glucose tolerance during an HFD-induced regain phase
Natural herbal medicines	Rhizoma Coptidis alkaloids (He et al., 2016)	High-fat, high-cholesterol diet-fed mice	*Sporobacter termitidis*, *Alcaligenes faecalis*, and *Akkermansia muciniphila*	*E. coli*, *Desulfovibrio* C21_c20, and *Parabacteroides distasonis*	Reduced weight gain and TC, TG, LDL-C, and TBA
	Herbal formula (Tong et al., 2018)	Patients with T2DM and hyperlipidemia	*Blautia Faecalibacterium* spp.	*Alistipes*, *Oscillibacter*, and *Bacteroides*	Improved HOMA-IR and plasma triglyceride levels,
	*Hirsutella sinensis* (Wu et al., 2019)	HFD mice	*Parabacteroides goldsteini*	*Mucispirillum schaedleri*, *Shewanella*, and algae	Improved insulin resistance and lipid metabolism
	Berberine (Sun et al., 2017; Wang et al., 2017)	ob/ob mice, WT and FXRint-/- mice	*Enterobacter* and *Escherichia−Shigella*	Firmicutes and Bacteroidetes	Reduced weight gain and plasma and liver lipids. Improved insulin resistance
	Pu-erh tea (Huang et al., 2019)	HFD mice and patients with hyperlipidemia	Bacteroidia	Lactobacillus, Bacillus, Enterococcus, Lactococcus, Streptococcus, and Leuconostoc genera	Reduced weight gain and serum TC and TC
	Blueberry extract (Guo et al., 2019)	C57BL/KsJ db/db mice and HFD mice	Akkermansia and Bifidobacterium	Desulfovibrio and Bilophia	Promoted the activation of BAT and the browning of WAT and improved lipid metabolism in the liver and adipose tissue

### Prebiotics

Prebiotics are organic substances that are not digested and absorbed by the host but selectively promote the metabolism and proliferation of beneficial bacteria in the body, thereby improving host health. After reaching the colon, prebiotics are decomposed and utilized, promoting the growth of colonic microbiota, where they play an important role in improving the intestinal microecology and regulating lipid metabolism. Additionally, prebiotics can improve lipid metabolism by boosting probiotics and producing SCFAs (Yu et al., 2021). Different types of prebiotics include isomalto-oligosaccharide, fructooligosaccharide, galacto-oligosaccharide (GOS), xylooligosaccharide, lactulooligosaccharide, soybean oligosaccharide, and inulin (Slavin, 2013). Inulin-type fructans regulate gut microbiota activities and reduce cholesterol and triglycerides (Delzenne and Williams, 2002). Different types of lactose-derivatives such as GOS, lactosucrose, and tagatose can effectively inhibit the expression of fat-forming genes and enzymes, promote the combination of cholesterol and probiotics, reduce the cholesterol level, and promote the transformation of BA enzyme and lipid metabolism to regulate lipid level (Nath et al., 2018). Red gram prebiotics can reduce the TG, TC, and HDL-C levels in hyperlipidemia-model rats (Shakappa et al., 2018). By changing the composition of gut microbiota through prebiotics, the mediated effect of GLP-2 can improve the intestinal-barrier function, reduce the amount of endotoxin into the blood, and improve the metabolism of blood lipids (Cani et al., 2009). A meta-analysis and a study supports that inulin can modulate lipid metabolism by regulating gut microbiota (Liu et al., 2017; Li et al., 2019). A clinical trial has shown that prebiotics can increase breath-hydrogen excretion (a marker of gut-microbiota fermentation) by approximately threefold, increase plasma GLP-1 and PYY concentrations, and lower hunger rates (Cani et al., 2009). Interestingly, some juice and tea extracts are also considered to have prebiotic effects. A controlled clinical trial has shown that the daily intake of 300 mL of orange juice in 10 women can significantly improve blood lipids and increase the abundance of beneficial bacteria in the intestinal flora, suggesting that orange juice may act as a prebiotic (Fidélix et al., 2020). The EGCG3’’Me in oolong tea is a potential prebiotic, which ameliorates HFD-induced gut dysbiosis and significantly decreases the ratio of Firmicutes to Bacteroidetes (Zhang et al., 2018). Green tea extract combined with isomalto-oligosaccharides can effectively prevent HFD-induced obesity and fat accumulation in liver and muscle, effectively regulate liver metabolism related to lipid metabolism, and prevent the HFD-induced increase in LPS and pro-inflammatory factors in the circulatory system, which may be due to the increased abundance of beneficial bacteria, thereby restoring the ratio of Firmicutes to Bacteroides and increasing the ratio of Prevotella to Bacteroides (Singh et al., 2017). In summary, the mechanism by which prebiotics improve the body’s metabolism may be to improve the composition of the gut microbiome, improve the intestinal barrier function, and show a potential beneficial effect on the regulation of lipid metabolism.

### Probiotics

Probiotics is a general term for microorganisms that can improve the microecological balance of the host and play a beneficial role in it. Probiotics such as *Bifidobacterium*, *Lactobacillus*, and yeast can regulate appetite, obesity, and the cardiovascular system (Cheik et al., 2008). Probiotics in the intestine can balance the amount of metabolites (BAs, LPS, and SFCAs) in the intestine, which can reduce liver cholesterol synthesis and, consequently, the blood lipid content (Repa et al., 2002; Chiang, 2009; Kim et al., 2014). Mixed feeding of probiotics can reduce the levels of TG, TC, and fatty acids in hyperlipidemic hamsters (Stancu et al., 2014). Interestingly, single strains have the same effect. Animal studies has shown that *Lactobacillus reuteri* 263, *Lactobacillus fermentum* MTCC: 5898, and *Lactobacillus casei* YBJ02 are potential probiotics that can reduce the level of blood lipids in the body (Huang et al., 2015; Qian et al., 2019; Yadav et al., 2019). Butyrate-producing probiotics also induce peroxisomal activity and improve lipid metabolism (Weng et al., 2015). Consistent with animal experiments, probiotic supplementation also significantly reduces serum TC and LDL-C in patients with hyperlipidemia (Sharma et al., 2016). A 12-week randomized, double-blind, placebo-controlled clinical trial has shown that the combination of three *Lactobacillus plantarum* strains can reduce blood cholesterol in patients (Guerrero-Bonmatty et al., 2021). Additionally, a study involving 70 healthy adults has demonstrated that the daily supplementation of *Lactobacillus plantarum* Q180 can significantly improve blood-lipid indicators such as LDL-C and TC after meals by adjusting the composition of the intestinal flora (Park et al., 2020). These studies reveal that probiotics can improve the level of TC, TG, HDL-C, and LDL-C by promoting the growth of probiotics, regulating related metabolites.

### Fecal Microbiota Transplantation

FMT is the process of transferring functional bacteria in the feces of healthy people to the intestinal tract of patients in a certain way to regulate gut microbiota. The goal of FMT is to restore the healthy diversity of gut microbiota and provide effective treatment for diseases inside and outside the intestinal tract. FMT has been successful in treating *Clostridium difficile* infections in several trials (Kao et al., 2017). In recent years, FMT has also been supposed as a potential therapy to improve lipid metabolism (Lai et al., 2018). The transplantation of fecal bacteria from healthy mice to diet-induced obese mice improves the gut microbiota composition, function, and metabolism of obese mice (Sun et al., 2019). An animal study has found that the transplantation of resveratrol–microbiota to HFD mice can modulate lipid metabolism, stimulate the development of beige adipocytes in WAT, reduce inflammation, and improve intestinal-barrier function (Wang et al., 2020). In a new study published in *Gastroenterology* (Rinott et al., 2021), 90 patients with abdominally obese or dyslipidemia have been randomly given three different dietary interventions for 6 months. After the intervention, the patients’ own fecal microbiota has been made into capsules for subsequent FMT. Results show that autologous FMT could significantly inhibit the rebound of body weight, waist circumference, and insulin level.

### Natural Herbal Medicines

Increasing evidence shows that several natural herbal medicines can effectively improve dyslipidemia by regulating gut microbiota. Rhizoma Coptidis (RC) alkaloids can reduce the body-weight gain and serum TC, TG, and LDL-C levels of B6 mice. RC alkaloid feeding significantly increases the abundance of *Sporobacter termitidis*, *Alcaligenes faecalis*, and *A. muciniphila* while suppressing the abundance of *E. coli*, *Desulfovibrio* C21_c20, and *Parabacteroides distasonis* in mouse gut (He et al., 2016). Berberine may exert its lipid-lowering by promoting the production of butyrate and inhibiting the activity of BSH in gut microbiota, significantly increasing the levels of tauro-conjugated bile acids, and inhibit ileal FXR signal transduction pathway (Sun et al., 2017; Wang et al., 2017). A multicentric randomized clinical trial has shown that an herbal formula consisting of eight herbs may ameliorate type-2 diabetes with hyperlipidemia by enriching beneficial bacteria, such as *Blautia* and *Faecalibacterium* spp (Tong et al., 2018). Polysaccharides isolated from *Hirsutella sinensis* can promote the growth of specific intestinal bacteria, such as *Parabacteroides goldsteinii*, thereby improving obesity and adipose tissue thermogenesis, enhancing intestinal integrity and reducing levels of inflammation and insulin resistance. in mice (Wu et al., 2019). Theabrownin from Pu’er tea and blueberry extracts can reshape the intestinal flora of HFD mice, regulate BA pools, and activate BA receptors to lower cholesterol (Guo et al., 2019; Huang et al., 2019).

## Prospect

Overwhelming evidence shows that changes in the abundance and composition of gut microbiota are generally linked to numerous hyperlipidemia-related diseases, including obesity, diabetes, arteriosclerosis, stroke, coronary heart disease, and myocardial infarction, among others. With the development of sequencing technology and the application of GF models in recent years, research on gut microbiota and microbiota-related metabolites has been greatly developed. Accordingly, the role and mechanism of gut microbiota in hyperlipidemia development have also been gradually revealed. New therapeutic approaches that target gut microbes for the treatment and prevention of hyperlipidemia represent exciting areas of investigation. Gut microbiota participates in the occurrence and development of hyperlipidemia by regulating the storage, decomposition, and distribution of lipids. Targeted therapy based on gut microbiota can effectively improve hyperlipidemia and its complications. The association and extensive plasticity of gut microbiota and microbiota-related metabolites with hyperlipidemia makes gut microbiota a potential marker for disease prediction and diagnosis, as well as a target for disease prevention and treatment. These treatments include probiotics, prebiotics, or FMT, among others, which may be used in the future to terraform the microbial community and alter its functional output for the betterment of the host. With increasing awareness of the relationship between the gut microbiome and hyperlipidemia-related diseases, we can hold high expectations for the clinical application of gut-microbiome modulation.

## Author Contributions

Conceptualization, XJ and WX. Funding acquisition, WX and SW. Methodology, XL and LZ. Project administration, XJ and SW. Writing – original draft, XJ and WX. Writing – review and editing, RW and SW. All authors contributed to the article and approved the submitted version.

## Funding

This work was supported by the National Natural Science Foundation of China (81773956, 81872990, and 82004149), the Provincial Natural Science Foundation of Fujian (2019I0016), and the Young and Middle-aged Backbone Program of Fujian Provincial Health Commission (2018-ZQN-65).

## Conflict of Interest

The authors declare that the research was conducted in the absence of any commercial or financial relationships that could be construed as a potential conflict of interest.

## Publisher’s Note

All claims expressed in this article are solely those of the authors and do not necessarily represent those of their affiliated organizations, or those of the publisher, the editors and the reviewers. Any product that may be evaluated in this article, or claim that may be made by its manufacturer, is not guaranteed or endorsed by the publisher.
